# Restless Legs Syndrome and Neurological Comorbidities: A Narrative Review

**DOI:** 10.3390/jcm15103725

**Published:** 2026-05-12

**Authors:** Kyrillos Ghattas, Helen Hernandez, Yongwoon Huh, Zhanna Fast, Zhikui Wei

**Affiliations:** 1 Jefferson Sleep Disorders Center, Thomas Jefferson University Hospital, Philadelphia, PA 19107, USA; kyrillos.ghattas@jefferson.edu (K.G.); yongwoon.huh@jefferson.edu (Y.H.); zhanna.fast@jefferson.edu (Z.F.); 2 Department of Psychiatry, Thomas Jefferson University Hospital, Philadelphia, PA 19107, USA; 3 Department of Neurology, Thomas Jefferson University Hospital, Philadelphia, PA 19107, USA; helen.hernandez@jefferson.edu; 4 Department of Internal Medicine, Thomas Jefferson University Hospital, Philadelphia, PA 19107, USA

**Keywords:** restless legs syndrome, neurological disorders, Parkinson’s disease, multiple sclerosis, migraine, stroke, dementia, peripheral neuropathy

## Abstract

Restless legs syndrome (RLS) is a common yet underrecognized neurological disorder characterized by uncomfortable sensations and an irresistible urge to move he legs, typically following a circadian pattern. RLS frequently co-occurs with various other neurological diseases, raising questions about shared mechanisms and clinical consequences. This review synthesizes evidence on the prevalence, outcomes, and pathophysiology of RLS in various neurological disorders, including Parkinson’s disease, multiple sclerosis, migraine, dementia, stroke, epilepsy, and peripheral neuropathy. In Parkinson’s disease, RLS is linked to disease progression and dopaminergic therapy. In stroke and multiple sclerosis, RLS is associated with structural lesions at specific locations, such as the pons or spinal cord. In epilepsy, RLS is associated with refractory or nocturnal seizures. In neuropathies, disruption of small sensory fibers may contribute to RLS symptoms. In dementia, RLS adds diagnostic complexity. Overlapping mechanisms between RLS and its neurological comorbidities include altered sensorimotor processing, brainstem and spinal circuitry, and sleep/arousal regulation. RLS in neurological conditions often worsens sleep quality, mood, and fatigue, and contributes to reduced quality of life and worse outcomes. Future research should prioritize longitudinal designs, standardized diagnostic approaches, and mechanistically driven studies to clarify relationships between RLS and these neurological comorbidities.

## 1. Introduction

Restless legs syndrome (RLS) is a common yet often underrecognized neurological disorder marked by an irresistible urge to move the legs, usually accompanied by uncomfortable sensations, such as tingling or crawling, that worsen at rest, intensify in the evening or night, and improve with movement [1]. RLS frequently coexists with various neurological disorders, prompting a growing interest in understanding the shared neurobiological pathways and clinical consequences of these associations [2].

Several mechanisms overlap between RLS and neurological diseases such as Parkinson’s disease. These include dysregulation of dopaminergic circuits, altered brain iron levels, and changes in brain structure and connectivity [3]. In RLS, it is thought that the brain iron deficiency, particularly in the substantia nigra (SN) and other subcortical regions, contributes to dopamine dysregulation and subsequent activation of sensorimotor symptoms [4]. The dysfunction of the dopaminergic system has been implicated in conditions such as dementia [5], migraine [6], and Parkinson’s disease [3], and may contribute to the associations of these conditions with RLS. In addition, central lesions such as pontine stroke [7] or spinal demyelinating plaques [8], and peripheral small-fiber neuropathies [9], have all been associated with RLS symptoms.

The diagnosis of RLS in neurological conditions can be challenging because of the overlapping sensory and motor disturbances. For example, neuropathy and RLS can both present with tingling, crawling, or shock-like leg sensations [10], and Parkinson’s disease and RLS both have motor restlessness [11]. Moreover, because RLS diagnosis relies on self-reported symptoms, accurate diagnosis may be difficult in patients with cognitive impairments, which is common in neurological diseases. Indeed, an alternative diagnostic approach has been proposed in these patients utilizing observed signs and symptoms [10]. Finally, RLS may emerge before or after the onset of a neurological disorder or its treatment, further complicating diagnostic clarity and underscoring the complex interplay between RLS and these neurological conditions.

Several conditions commonly mimic RLS and should be carefully differentiated. Peripheral neuropathy typically presents with persistent sensory symptoms that may not demonstrate circadian variation and are not consistently relieved by movement [12]. Akathisia is characterized by generalized inner restlessness without a sensory component and lacks the circadian pattern seen in RLS [12]. Nocturnal leg cramps are usually painful, focal, and associated with muscle tightening rather than an urge to move. Positional discomfort and arthritis-related pain are also common mimics but are typically localized and not associated with the characteristic relief with movement seen in RLS [12]. Careful history-taking focused on symptom timing, triggers, and response to movement remains essential for distinguishing true RLS from these mimics.

This review provides an updated overview of the epidemiology, clinical characteristics, potential pathophysiological mechanisms, and practical management considerations of RLS across common neurological conditions, with emphasis on shared themes across disorders (Table 1). We discuss RLS across a range of disease categories, including neurodegenerative disorders (e.g., Parkinson’s disease and dementia), vascular conditions (e.g., stroke), neuroinflammatory conditions (e.g., multiple sclerosis), and other neurological disorders such as migraine, epilepsy, and peripheral neuropathy, recognizing the heterogeneous nature of these conditions. Less common neurological disorders, such as amyotrophic lateral sclerosis, have also been associated with RLS [13,14] but are beyond the scope of this review. RLS often coexists with other sleep disturbances, such as insomnia and sleep-disordered breathing, which are common in neurological populations. However, this review focuses on RLS given its distinct sensorimotor features and management considerations.

## 2. Search Strategy

We conducted a literature search using PubMed to identify primary research articles and systematic reviews examining RLS in association with neurological disorders. The search included studies published in English, primarily within the last 25 years, and was limited to human subjects. We included clinical studies, randomized controlled trials, systematic reviews, and meta-analyses. To ensure relevance, we restricted results to articles with RLS or its synonyms in the title and at least one common neurological condition (e.g., Parkinson’s disease, multiple sclerosis, stroke, and epilepsy) also in the title. We excluded case reports, case series, and studies focused solely on periodic limb movements (PLMs) or periodic limb movement disorder (PLMD). Additional relevant references were included through the review of references in the retrieved articles and review of major clinical guidelines on RLS. Well-cited articles providing context information on each neurological condition and RLS were also obtained through a PubMed search. A total of 178 articles were obtained through this search strategy, and 108 were included in this review. Evidence appraisal was conducted qualitatively, with emphasis on sample size, study design, diagnostic rigor, and risk of bias. Greater weight was given to systematic reviews and meta-analyses, clinical guidelines, and high-quality clinical studies. This review primarily focuses on adult populations, as most available studies of RLS in neurological disorders have been conducted in adults.

## 3. RLS and Neurological Comorbidities

### 3.1. RLS and Parkinson’s Disease

Parkinson’s disease (PD) is a progressive neurodegenerative condition that results in striatal dopamine depletion and malfunction of basal ganglia circuits. The loss of dopaminergic input creates an imbalance between the direct and indirect motor pathways, leading to the hallmark motor symptoms of PD, including resting tremor, bradykinesia, rigidity, and postural instability [41]. RLS and PD both involve perturbations of dopaminergic systems, brain iron dysregulation, and dopaminergic responsiveness [2]. However, epidemiologic evidence for a close association between these two conditions remains unclear.

Several studies in drug-naïve patients found no significant difference in RLS frequency between PD patients and controls, including Calzetti et al. [42], Angelini et al. [43], and a multicenter case–control study in Japan [11]. In contrast, multiple clinic-based series and meta-analyses pooling treated and mixed-stage PD populations reported a higher prevalence of RLS in PD, ranging from 11.9% to 25%, and exceeding that in controls [15,16,17,18,19,20]. Longitudinal data can potentially help reconcile some of the discrepancies, as in de novo cohorts followed prospectively, incident RLS often developed after diagnosis of PD within months to a few years of starting dopaminergic therapy, supporting the view that RLS develops in PD possibly secondary to disease evolution and/or treatment [44,45]. This view is also consistent with the observation that RLS prevalence is generally higher than expected in patients with treated PD compared with drug-naïve PD [2].

Findings from newer systematic reviews and meta-analyses have added additional complexity and nuances. The two recent meta-analyses by Yang et al. and Maggi et al. both estimated a higher prevalence of RLS in PD than that in healthy controls [21,46]. However, the first study also showed important demographic differences. The prevalence of RLS in PD is lower in Asia than outside of Asia, and higher in females than in males. It also showed a higher prevalence of RLS in treated PD compared with untreated PD [46]. In contrast, the study by Maggi et al. showed that the occurrence of RLS in PD was not significantly modified by parameters related to dopaminergic therapy, such as dose and duration [21]. This observation could be consistent with a double-sided role of dopaminergic therapy in PD, which can initially relieve RLS symptoms and later induce or exacerbate RLS through the augmentation phenomenon [21].

Current evidence supports an association, but not definitive causality between PD and RLS. Rates of RLS are not consistently elevated in untreated PD, arguing against shared causative biology alone [11,42,43]. Increased prevalence and incidence after PD onset and treatment suggest that RLS may develop secondary to PD progression and/or dopaminergic therapy [21,46]. But heterogeneity across studies in terms of study design, PD stage, medication status, diagnostic rigor, population ethnicity, and sample source (community vs. specialty clinic) prevents establishing a clear causal pathway [21,46].

Several observational studies also reported higher incident PD in patients with RLS, suggesting a possible bidirectional relationship between RLS and PD. In a large cohort of US veterans, prevalent RLS was associated with a higher risk of incident PD over 8 years of follow-up [47]. Similar findings were observed in a prospective cohort of male health professionals and in a Korean National Health Insurance Service Sample Cohort [48,49]. It was thought that RLS could be either an early manifestation or a potential risk factor for PD [48,49]; however, alternative explanations, such as diagnostic misclassification and surveillance bias, must also be considered [50].

Despite an unclear relationship between RLS and PD, RLS is linked to a greater disease burden and increased complexity of PD symptoms. More specifically, RLS in PD is associated with greater non-motor PD symptoms such as depression, anxiety, fatigue [21,51], and poor sleep quality [15,17,52], and higher bodily discomfort and pain scores [19,20]. It is also linked to higher rates of impulse disorders [53] and more severe cognitive and autonomic dysfunctions [21]. Collectively, these features of RLS in PD can complicate management and contribute to poor quality of life in these patients.

The pathophysiologic mechanisms between RLS and PD remain unclear. Some observations suggest dopaminergic involvement, as both conditions respond to dopaminergic medications and exhibit changes in the striatal dopaminergic circuits [3]. However, the disease mechanisms also differ significantly. RLS is characterized by brain iron deficiency and subsequent impairment in dopaminergic signaling [4], whereas PD involves iron accumulation and progressive nigrostriatal neuronal loss [3]. Nevertheless, it has been proposed that iron deposition in PD creates a dopaminergic-deficient environment in the brain through dopaminergic cell death and mitochondrial dysfunction, establishing a possible pathogenic substrate for RLS [3]. Given the strong association between RLS and the severity of non-motor symptoms of PD, involvement of non-dopaminergic systems, such as the noradrenergic system, has been suggested [51]. In addition, imaging studies have shown structural changes in the posterior cingulate cortex and altered resting state functional connectivity in the sensorimotor networks in PD patients with RLS, supporting a possible role for altered brain structure and connectivity [3].

Clinicians should recognize that RLS is more likely to emerge in treated or later-stage PD than at diagnosis and may represent a therapy-related phenomenon rather than a coincidental comorbidity [21,46]. To avoid misdiagnosis of RLS, it is essential to distinguish true RLS from PD-related mimics, such as motor restlessness that persists through movements or periodic leg movements during sleep (PLMS), which lack a sensory component [3]. Importantly, the emergence of RLS symptoms in PD should not automatically trigger dopaminergic escalation, as excessive dopaminergic exposure may worsen long-term control of RLS symptoms in susceptible patients. In such cases, α2δ ligands may be preferable given their lower risk of augmentation [3]. Further studies are needed to determine optimal treatment strategies for RLS in later-stage PD.

### 3.2. RLS and Multiple Sclerosis

Multiple sclerosis (MS) is a chronic demyelinating and degenerative disorder affecting the central nervous system. It involves immune-mediated destruction of myelin and axons in the brain and spinal cord and manifests with a wide range of neurological symptoms, including paresthesia, muscle weakness, visual impairment, and gait and balance problems [54].

Multiple studies have identified a significant association between RLS and MS. A systematic review and meta-analysis of RLS in MS by Schürks et al. in 2013 found a fourfold increase in the odds of RLS in patients with MS compared with those without [24]. A subsequent systematic review and meta-analysis in 2018 by Ning et al. estimated the pooled prevalence of RLS in MS to be higher in females than males (26% vs. 17%) [25]. An updated meta-analysis in 2023 included 75 studies, the largest to date, and estimated the pooled prevalence of RLS in MS and clinically isolated syndrome, a precursor condition to MS, to be 28% compared to 9% in controls. Prevalence was again higher in women than men (30% vs. 22%) [22].

Although recent meta-analyses have consistently reported a higher of risk RLS in the MS population, case–control studies using a strict diagnostic approach have yielded lower prevalence estimates than those reported in the meta-analyses. For example, a recent case–control study reported a 15% prevalence of RLS in an MS cohort and a 22% false-positive rate when RLS was diagnosed using a questionnaire without clinical confirmation by a sleep specialist [23]. Most false-positive diagnoses occurred in patients with leg stiffness, spasticity, and/or severe sensory symptoms, common MS manifestations that mimic motor and sensory disturbances in RLS [23]. These findings highlight the heterogeneity in diagnostic methodologies across studies, which likely contributes to ascertainment bias.

There are significant differences in clinical presentation between those with MS and RLS and those with MS alone. Compared with patients with MS alone, those with both conditions are older, have greater disability, are more likely to have primary progressive MS- the most common type of MS, and report more sleep-related complaints, including insomnia and excessive daytime sleepiness [8,23,55]. Predictors of RLS in MS vary across studies and may include greater disability on the Expanded Disability Status Scale (EDSS), infratentorial/spinal lesions, family history of RLS, and pyramidal tract involvement [8,23].

The pathophysiologic links connecting RLS and MS have not been clearly defined. Some hypotheses have indicated disruption of spinal cord structures in MS, including disruption of the corticospinal tract [24], increased spinal cord excitability [56], and altered dopaminergic innervation [24,25]. In addition, medullary hyperexcitability caused by demyelinating plaques in the pyramidal tract or lesions in the central dopaminergic pathways may contribute to the development of RLS in MS as well [23].

Clinicians should screen for RLS in MS patients who report poor sleep quality or excessive daytime sleepiness. Those with a family history of RLS, severe disability, infratentorial or spinal lesions, or pyramidal tract involvement are at higher risk [8,23]. Management of RLS in MS should generally follow standard RLS treatment principles [57]. However, adverse effects of commonly used medications for RLS, such as sedation, dizziness, cognitive impairment with α2δ ligands, and neuropsychiatric symptoms with dopamine agonists, are relatively common and require careful monitoring in MS patients.

### 3.3. RLS and Migraine

Migraine is a chronic neurological condition characterized by throbbing headaches associated with symptoms such as photophobia, phonophobia, nausea, and vomiting [58]. It is one of the leading causes of global disability based on years lived with disability [59]. The pathophysiology of migraine is complex and involves activation and sensitization of the trigeminovascular systems and dysfunctional sensory processing within the brainstem and diencephalic networks [60].

Some studies have reported a significant association between migraine and RLS. In a retrospective cohort study done in Taiwan involving 23,641 patients with migraine and 94,564 subjects without migraine, the risk of RLS was 1.42-fold higher in the migraine cohort than in the non-migraine cohort [61]. An observational and cross-sectional study done in Europe showed that RLS is more prevalent in patients with migraine than in controls (16.9% vs. 8.7%) [27]. Recent meta-analyses of observational studies estimated the prevalence of RLS in adults with migraine to be significantly higher than in the general population [26,62]. Of note, the association between RLS and migraine has also been shown in children and adolescents, in which the frequency of RLS was found to be 22% in those with migraine compared with 5% in the controls [63].

Despite relatively consistent findings of a higher RLS prevalence in migraine, several methodological caveats across studies warrant consideration. First, there is substantial heterogeneity in diagnostic approaches for RLS across studies. For example, the meta-analysis by d’Onofrio included studies using different versions of the International RLS Study Group (IRLSSG) diagnostic criteria [1,10] and questionnaire-based methods [64,65]. This variability may contribute to the misclassification of RLS and influence the prevalence estimates [26]. Differences in demographics may further contribute to the variability, as RLS prevalence is higher in Europe and North America and lower in Asia [66]. Indeed, a study from Taiwan showed an annual RLS incidence rate of 3.52/10,000 person-years in the control cohort, which was lower than that of a German cohort (9–22/1000 person-years) [61]. Such regional variations may also influence the estimates of RLS risk among patients with migraine.

RLS in migraine appears to be more severe and associated with poorer sleep quality compared with RLS in the control group [27]. Persistent RLS in migraine during follow-up has also been linked to greater depressive symptoms and headache-related disabilities [67]. The risk of RLS appears higher in older patients and in patients with longer migraine duration and greater migraine-related disability [26,61]. Additional predictors for RLS in migraine include a positive family history of RLS, more severe depressive symptoms, greater daytime sleepiness, and higher serum phosphorus levels [68].

The mechanistic links between RLS and migraine remain unclear. Some studies suggest the role of dopamine-mediated pathways, supported by a few observations such as relief of migraine attacks by dopaminergic antagonists and the presence of dopamine-mediated premonitory symptoms in migraine, such as yawning, fatigue, and mood changes [6,69]. Decreased brain iron levels may present another plausible mechanism. Greater iron depletion in the periaqueductal gray has been observed in migraine, possibly related to the brain iron deficiency in RLS [26]. Genetic factors may also contribute, as genetic variants of MEIS1 have been associated with a higher risk of RLS in migraine [70].

Migraine patients with more severe headache symptoms, greater disability, depression, and sleep disturbance should be screened for RLS [67]. Patients should be screened both at baseline and during follow-up, as RLS risk increases with time in the migraine population [67]. Evaluation should prioritize reversible contributors, including iron deficiency and medication triggers. Notably, medications often prescribed for migraine—such as antidepressants (e.g., venlafaxine and duloxetine), dopamine-blocking antiemetics (e.g., metoclopramide), and sedating antihistamines (e.g., diphenhydramine)—may precipitate or worsen RLS symptoms [57]. Patients with a known diagnosis of or risk factors for RLS should be counseled and monitored carefully.

### 3.4. RLS and Dementia

Dementia is a clinical syndrome characterized by a persistent and progressive decline in cognitive domains that leads to impaired social functioning and reduced ability to perform daily tasks. It is frequently associated with more than one neuropathology, most commonly a combination of Alzheimer’s disease (AD) and cerebrovascular pathology. Despite advances in understanding of its pathophysiology, treatment options for dementia remain limited, posing great challenges for patients and families affected by this condition [71].

The studies on the association between RLS and dementia are limited. Some observational studies suggest a higher risk of incident dementia in patients with RLS. A large nationwide retrospective cohort study in Korea reported an increased risk of incident all-cause dementia (adjusted hazard ratio of 1.46, 95% confidence interval (CI) [1.24–1.72]) in patients aged ≥ 60 years with RLS compared with controls [30]. These findings contrast with earlier cross-sectional studies that showed mixed results regarding cognitive function in patients with RLS, with some showing impairment in frontal lobe function, while others reported no cognitive impairment or better cognitive performance than sleep-deprived controls [72,73,74,75,76]. Data on the prevalence of RLS in patients with dementia are also limited and variable. A cross-sectional study from Italy examined RLS in a cohort of patients with AD using standard RLS diagnostic criteria and reported a prevalence of 4% [28]. In contrast, a study from Brazil reported the prevalence of RLS to be 15.7% in a cohort of adults with mixed dementia [29].

There are important limitations in the methodology of these studies. Kim et al. provide evidence suggestive of a possible temporal relationship between RLS and incident dementia using a nationwide, retrospective, longitudinal cohort design. Still, reliance on administrative coding likely introduces exposure misclassification and detection bias [30]. The mean follow-up of four years may have been insufficient for causal inference. In contrast, clinic-based studies in dementia populations [28,29] allow closer clinical characterization; however, the diagnosis of RLS using self-reported symptoms may be less reliable in AD and other dementias. This approach creates competing risks of false positives and false negatives. In addition, neither study includes a control group [28,29], limiting the interpretation of how these prevalence estimates compare with those in the general population.

RLS has been associated with worse neuropsychiatric symptoms in dementia from observational data. For example, in patients with AD, patients with RLS were reported to be more apathetic while having similar performance on the mini-mental status exam (MMSE), activities of daily living (ADL), and instrumental activities of daily living (iADL) compared with those without RLS [28]. RLS in AD may also manifest as nighttime agitation, characterized by behavioral disturbances such as aggression or wandering [77]. In another cohort of dementia patients, those with RLS were reported to have greater sleep disturbance in addition to frequent neuropsychiatric symptoms [29]. In terms of risk of dementia in RLS patients, one study suggested a higher risk of vascular dementia compared to AD, though this finding requires further validation [30].

The mechanistic links between RLS and dementia remain incompletely understood. The elevated risk of incident dementia in RLS was hypothesized to be partially mediated by sleep disturbances [30], which have been linked to AD pathology, including amyloid deposition [78]. In addition, an autopsy study of brains from patients with RLS showed increased silent microvascular disease with gliosis, suggesting a possible contribution of vascular injury to conditions such as dementia [79]. Possible pathophysiological mechanisms between RLS and dementia could also include impaired dopaminergic transmission and altered brain iron. Both AD and RLS have shown decreased dopamine receptors in different parts of the brain [30], although brain iron accumulation is more commonly linked to AD pathology, different from brain iron deficiency in RLS [4,31].

In patients with dementia, screening for RLS should be proactive because symptoms are frequently underreported. Screening for RLS should be prioritized for patients with apathy, neuropsychiatric symptoms, sleep disturbance, and nighttime agitation [77]. Given the impaired cognitive function associated with dementia, clinicians should obtain collateral history from caregivers and incorporate supporting evidence such as family history or iron deficiency. Evaluation should prioritize reversible contributors, particularly medication triggers. Medications such as sedating antihistamines and serotonergic antidepressants, which are known to exacerbate RLS symptoms, have been linked to nighttime agitation in patients with RLS and dementia.

### 3.5. RLS and Stroke

Stroke is the second leading cause of death and long-term morbidity worldwide [80]. It is characterized by neurological deficits resulting from focal injury to the central nervous system caused by a vascular event [81]. Stroke can be broadly classified into ischemic stroke and hemorrhagic stroke [81]. Despite different mechanisms, ischemic and hemorrhagic stroke share many common risk factors—such as hypertension, diabetes, hyperlipidemia, and smoking—and often lead to overlapping neurological impairments [82].

Current literature has shown inconsistent findings regarding an association between RLS and stroke. Smaller case–control and cohort studies have shown a significant association between the two. For example, a case–control study involving hospitalized stroke patients reported higher odds of RLS in stroke patients compared with controls after adjusting for vascular and RLS risk factors [83]. Similarly, ~10% of patients in a cohort of 346 stroke patients fulfilled standard diagnostic criteria for RLS, and pre-existing RLS in this cohort was associated with subcortical stroke [32]. In contrast, a meta-analysis of 8 eligible studies with a total of > 600,000 patients did not find a higher risk of cardiovascular or cerebrovascular events in patients with RLS [84].

One important factor to consider when interpreting these results is the effect of confounding. RLS is associated with common vascular risk factors, such as hypertension, diabetes, atherosclerosis, and obesity [85,86,87]. The variable prevalences of these risk factors may contribute to heterogeneous findings across studies on RLS and stroke risk, and incomplete adjustment for them may lead to residual confounding. Indeed, in the meta-analysis by Katsanos et al., RLS patients were found to have a higher risk of cerebrovascular ischemia than controls in the unadjusted analysis, but not in the adjusted analysis for potential confounders [84].

Temporal associations between RLS and stroke have been reported, although the directionality remains uncertain. In the Caerphilly cohort, older men with frequent RLS symptoms had a higher risk of developing ischemic stroke over time [88]. However, a formal diagnosis of RLS was not established in this study, and it remains unclear how many met the diagnostic criteria of RLS. Similarly, a prospective study of stroke patients showed that among a subset of stroke patients who met criteria for RLS, RLS onset preceded stroke by 60 +/− 40 months [32]. Conversely, RLS has also been described after a stroke. A systematic review showed that RLS most commonly developed after brainstem stroke, particularly pontine lesions [7]. It can also occur with stroke in other locations, including the lentiform nucleus and thalamus [89].

The possible mechanistic links between RLS and stroke are not well established. RLS in patients with stroke has been associated with greater arterial stiffness, which may indicate greater vascular injury [90]. Long-term RLS has been associated with a higher burden of silent cerebral small vessel disease, which is a risk factor for stroke [91]. In RLS occurring after stroke, disruption of motor and sensory structures, such as the corticospinal or reticulospinal tracts, or dopaminergic dysregulation, may play a role [7,83]. Indeed, an imaging study found that patients with lenticulostriate infarction exhibited a hyperdopaminergic state in the lesioned striatum, which may be associated with RLS symptoms [33].

Patients with subcortical and brainstem stroke may warrant targeted evaluation for RLS. RLS developing after stroke has been associated with greater disability, slower functional recovery, poorer sleep quality, and greater fatigue [90,92]. These observations highlight the need for further research to determine whether early recognition and management of RLS could favorably influence recovery trajectories and functional outcomes in this population.

### 3.6. RLS and Epilepsy

Epilepsy is a chronic condition characterized by a perpetuating predisposition to epileptic seizures [93]. It is a common neurological condition and a leading cause of disability from a neurological disease based on disability-adjusted life years worldwide [94]. Although the pathophysiology of epilepsy is diverse across different epileptic syndromes, common mechanisms also exist, including increased neuronal excitability and synchronicity, often driven by ion channel mutations, synaptic dysfunction, and imbalances between excitatory and inhibitory neurotransmitters [95].

Current literature has shown a potential association between RLS and epilepsy. A systematic review and meta-analysis demonstrated that the risk of RLS is higher in adult patients with epilepsy compared to the control group (odds ratio of 2.09, 95% CI [1.53–2.85]) [34]. The pooled prevalence of RLS in adults with epilepsy was 14.9%, higher than that in the control group, although the prevalence estimate varied significantly across different studies [34]. For example, Shen et al. report a 3% prevalence of RLS in a cohort of epilepsy patients in China [35]. In contrast, in a cohort of patients with temporal lobe epilepsy (TLE), 42% of the patients with right-sided TLE and 15% of those with left-sided TLE had RLS, corresponding to an overall prevalence of 28.5% [36].

It is important to consider the methodological limitations of these studies when interpreting these findings. First, there is substantial heterogeneity in the diagnostic methods for RLS among these studies. Studies using standard IRLSSG criteria generally yielded lower prevalence estimates than those using single-question or questionnaire-based assessments (10.7% vs. 21.1%) [34]. Further, the use of antiseizure medications may contribute to the variability in the reported prevalence, as several antiseizure medications, particularly α2δ ligands, can treat RLS and may mask RLS symptom severity. Lastly, patient demographics may contribute to variations, as the prevalence of RLS in epilepsy was lower in Asian countries than in other regions [34].

Among different seizure types, RLS was more frequently associated with refractory seizures and nocturnal seizures [96]. In addition, among patients with TLE, the odds of having RLS are higher in those with right TLE than in those with left TLE. This observation may be related to the hypermotor activity seen in right TLE [36]. Overall, RLS in epilepsy is linked to inferior sleep quality, excessive daytime sleepiness, and lower quality of life [96].

The pathophysiological link between RLS and seizures is unknown. Some studies have suggested that decreased brain iron levels could play a role. Patients with medial TLE showed decreased iron levels in several subcortical structures, including the putamen, globus pallidus, substantia nigra, and red nucleus [37], which may serve as a substrate for the pathogenesis of RLS. Additionally, patients with TLE showed functional dopaminergic impairments affecting the nigrostriatal system [38], which may also increase the risk of RLS.

Clinicians should screen for RLS in patients with refractory or nocturnal epilepsy and those with TLE, although the higher risk of RLS in TLE has not been consistent across studies [96]. Evaluation for RLS is important in patients with epilepsy, as sleep impairment from RLS could increase the risk of epileptic attacks. Further, leg movements associated with RLS in patients with epilepsy could be mistaken for seizure activity. Careful evaluation for RLS in these patients may help prevent unnecessary escalation of antiseizure therapy and improve seizure control.

### 3.7. RLS and Peripheral Neuropathy

Peripheral neuropathy is characterized by nerve damage outside of the brain and spinal cord. It can affect different components of the peripheral nervous system, including the cranial nerves, spinal nerves and ganglia, nerve trunks and divisions in the upper and lower extremities, and the autonomic nervous system [97]. Peripheral neuropathy encompasses diverse syndromes from many different causes, such as diabetes, autoimmune diseases, nutritional deficiencies, and genetic mutations. It can lead to a variety of complications, contributing to significant morbidity and mortality [98].

A growing body of research has indicated an association between RLS and peripheral neuropathy. For instance, a systematic review and meta-analysis reported that ~21.5% of patients with peripheral neuropathy had RLS, and ~41.8% of patients with RLS also have peripheral neuropathy. Although these estimates are highly variable across different studies, both are significantly higher than rates observed in controls [39]. Studies have also evaluated RLS in specific peripheral neuropathy etiologies. RLS is more prevalent in diabetic neuropathy than in controls, with a pooled prevalence of approximately 25%. It is also frequently reported in several genetic and acquired neuropathies, including familial amyloid polyneuropathy, Charcot–Marie–Tooth disease, and uremic neuropathy, among others [99,100,101].

Important methodological limitations should be considered when reviewing these results. Studies incorporating formal IRLSSG criteria generally report lower RLS prevalence than those using less stringent definitions [39]. However, even with standard RLS diagnostic criteria, distinguishing RLS from neuropathy can remain uncertain, as there is potential symptomatic overlap between the two conditions, such as diurnal variations and restlessness/urge to move [12]. Further, prevalence estimates across studies vary depending on the diagnostic methods used for neuropathy. In uremic neuropathy, studies relying on retrospective chart review or clinical diagnosis of neuropathy reported higher RLS prevalence estimates than studies that performed electrophysiologic confirmation of neuropathy [101,102,103].

The clinical features of RLS appear to vary in different neuropathy syndromes. In diabetic neuropathy, patients with RLS were older and had higher pain scores than those without RLS [104]. In a cohort of patients with inherited and acquired neuropathies of mixed etiologies, those with RLS more commonly had a family history of RLS and were younger, with similar neuropathic symptoms between groups [105]. In a cohort of sensory axonal neuropathy, patients with RLS were older and had later-onset RLS, more frequent and severe RLS symptoms than controls with RLS [106].

The mechanistic links between RLS and neuropathy remain unclear. In diabetic neuropathy, RLS has been associated with small-fiber neuropathy, suggesting a possible role for small sensory fibers [9]. In painful polyneuropathy, nociceptive deafferentation may play a role in the pathogenesis of RLS [40]. In some hereditary neuropathies, it has also been hypothesized that the genes responsible for these neuropathies, such as hereditary sensory and autonomic neuropathy (HSAN), may also be expressed in the central nervous system and contribute to brain iron or dopamine dysregulation seen in RLS [105].

Given the substantial symptomatic overlap between RLS and neuropathy [12], clinicians should routinely screen for RLS in patients with neuropathy, especially those with sleep-onset symptoms, a family history of RLS, diabetes, renal disease, or iron deficiency. Careful history-taking is key to distinguishing RLS from neuropathy, and neurophysiologic studies such as nerve conduction studies/electromyography may be required to confirm neuropathy. When medication treatment is indicated, α2δ ligands are preferred since they are first-line therapy in both RLS and neuropathic pain [12]. Medications such as SNRIs or TCAs, which are commonly used for neuropathic pain, may worsen RLS and should be used cautiously in patients affected by both conditions [57].

## 4. Limitations of Current Literature

The current literature on RLS in neurological disorders is limited by several methodological challenges. A central issue is heterogeneity in diagnostic approaches, with many studies relying on questionnaire-based or existing diagnoses rather than applying standardized IRLSSG criteria [1,23,34,39]. Studies using stricter diagnostic confirmation consistently report lower prevalence rates than questionnaire-based studies, highlighting the impact of ascertainment bias [23,34,39]. Interpretation is also limited by study design and heterogeneity of study populations. Most available evidence is derived from cross-sectional and observational studies, which preclude causal inference and are susceptible to confounding [34,84,85,86,87]. Differences in the study population in disease stage, treatment exposure, and recruitment from specialty clinics versus community settings also contribute to inconsistent findings and reduced external validity [21,26,46].

## 5. Conclusions

RLS has variable associations with a range of neurological disorders. Increased prevalence in several neurological populations, including PD [21], MS [22], migraine [26], epilepsy [34], and peripheral neuropathy [39], has been reported. Additionally, RLS has also been shown to increase the risk of incident neurodegenerative conditions, including PD and dementia [30,47,48,49]. Across neurological conditions, RLS is associated with poorer sleep quality, depression, anxiety, fatigue, and reduced quality of life. It has also been linked to worse disease-related outcomes, including more severe non-motor symptoms in PD, greater disability in MS, refractory seizures in epilepsy, and slower stroke recovery [8,21,51,55,67,90,92,96].

**Figure 1:**
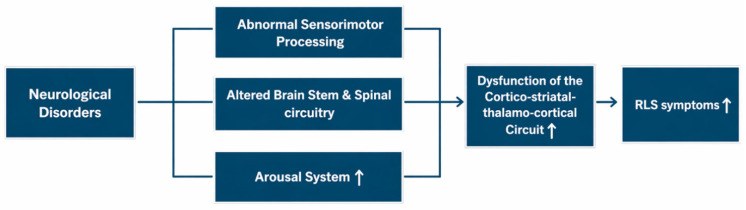
Proposed model for the mechanistic link between neurological disorders and restless legs syndrome (RLS). In neurological disorders, abnormal sensorimotor processing, altered brainstem and spinal circuitry, and increased arousal system activity may destabilize the cortico-striatal-thalamo-cortical (CSTC) circuit, thereby increasing RLS expression in susceptible individuals. The CSTC circuit refers to interconnected loops linking the cerebral cortex, striatum, and thalamus, and back to the cortex, which are involved in integrating sensory input, motor control, and behavioral regulation. White arrow indicates greater or increased activity/symptoms.

The mechanistic links between RLS and its neurological comorbidities remain incompletely understood. Across neurological disorders, associations with RLS may reflect disruption of shared pathways involved in sensorimotor processing, brainstem and spinal circuitry, and sleep/arousal regulation. Indeed, RLS has been conceptualized as a network disorder involving the dysfunction of the cortico-striatal-thalamo-cortical (CSTC) circuit and ascending arousal systems [107]. Within this framework, neurological disorders may not represent primary causes of RLS, but rather perturbations of shared sensorimotor networks and arousal systems. It is possible that lesions affecting basal ganglia dopaminergic systems, brainstem or spinal pathways, and peripheral sensory fibers may destabilize the CSTC circuit and lower the threshold for RLS expression in susceptible individuals (Figure 1). However, these proposed interactions are largely inferential and require further validation.

Clinicians should screen for RLS when patients with neurological disorders present with disproportionate nighttime or sleep-related sensorimotor complaints. The “URGE” mnemonic may be useful as a screening tool: urge to move, rest-induced, gets better with activity, and evening and night accentuation [108]. Even though symptoms such as spasticity, leg cramps, and neuropathic pain have partial symptomatic overlap with RLS, RLS tends to have a more distinct pattern [1]. Management of RLS in neurological disorders generally follows established principles of RLS treatment [57], including routine evaluation of iron status and minimization of exacerbating factors, such as caffeine intake, alcohol use, sleep apnea, and certain medications, including antihistamines, antidepressants, and antidopaminergic medications [57]. When a medication treatment is indicated for RLS, α2δ agents are likely first-line [57]. Dopamine agonists, commonly used in PD, may initially improve RLS symptoms but later worsen them through augmentation and should be carefully monitored [21].

Future studies should prioritize well-characterized longitudinal cohorts with standardized diagnostic criteria to better define incidence and temporal relationships between RLS and other neurological disorders. Improved methods to distinguish true RLS from common mimics in neurological populations are also needed, given the potential symptom overlap between RLS and neurological conditions. Mechanistic studies are needed to evaluate the roles of brain iron regulation, dopaminergic function, and network-level alterations across the cortical, subcortical, brainstem, and spinal structures. Greater attention should also be given to medication effects and treatment interactions, which may shape the clinical expression of RLS in disease-specific contexts. Addressing these priorities may improve diagnostic precision, support more targeted management, and ultimately improve outcomes in the neurological population affected by RLS.

## Figures and Tables

**Table 1:** RLS in Neurological Disorders.

Neurological Disorders	Prevalence *	Clinical Features of RLS in Neurological Disorders	Proposed Mechanisms
Parkinson’s Disease	11.9–25% [15,16,17,18,19,20,21]	Greater non-motor symptoms and neuropsychiatric symptoms of PD, including fatigue, depression, anxiety, sleep disturbance, autonomic dysfunction, and impulse control disorders; higher risk in females	Nigrostriatal dopaminergic degeneration; brain iron accumulation in basal ganglia structures; altered brain structure and connectivity [3]
Multiple Sclerosis	15–30% [22,23]	Older in age, greater disability, and more sleep-related complaints, including insomnia and excessive daytime sleepiness; Associations with infratentorial and spinal lesions	Disruption of central dopaminergic pathways secondary to demyelinating lesions involving spinal and brainstem circuits [24,25]
Migraine	16.9–22% [26,27]	More severe RLS symptoms and worse sleep quality; Greater depressive symptoms and headache-related disability; Higher risk in those with longer migraine duration and more severe migraine	Altered dopaminergic modulation within brainstem and diencephalic networks; regional brain iron depletion (e.g., periaqueductal gray) [6,26]
Dementia	4–15.7% [28,29]	Worse neuropsychiatric symptoms, such as apathy; Greater sleep disturbance; Possible manifestation as nighttime agitation; Association with vascular dementia	Reduced dopaminergic receptor activity in cortical and subcortical regions; cerebral iron accumulation associated with neurodegeneration; Increased microvascular disease and sleep disturbance [30,31]
Stroke	~10% (post-stroke cohorts) [32]	Greater disability, slower functional recovery, and worse sleep quality; Associations with pontine, lentiform, and thalamic lesions	Lesion-induced dopaminergic imbalance within motor and sensory pathways; disruption of corticospinal and reticulospinal tracts [7,33]
Epilepsy	3–28.5% [34,35,36]	Inferior sleep quality, excessive daytime sleepiness, and lower quality of life; Associations with nocturnal seizures and refractory seizures	Nigrostriatal dopaminergic impairment with reduced iron levels in subcortical nuclei in temporal lobe epilepsy [37,38]
Peripheral Neuropathy	21.5–41.8% [39]	Higher pain scores in diabetic neuropathy; More frequent and severe RLS symptoms	Small-fiber sensory dysfunction and nociceptive deafferentation affecting peripheral afferent pathways [9,40]

* The ranges of prevalence estimates from selected studies with heterogeneous methodologies, diagnostic criteria, and study populations were presented and should therefore be interpreted cautiously.

## Data Availability

No new data were generated in support of this review. All data discussed are derived from previously published studies cited in the manuscript.
